# Miniaturized and High Volumetric Energy Density Power Supply Device Based on a Broad-Frequency Vibration Driven Triboelectric Nanogenerator

**DOI:** 10.3390/mi15050645

**Published:** 2024-05-13

**Authors:** Liting Wu, Zewei Ren, Yanjun Wang, Yumin Tang, Zhong Lin Wang, Rusen Yang

**Affiliations:** 1 School of Advanced Materials and Nanotechnology, Xidian University, Xi’an 710126, China; wuliting269@163.com (L.W.); renzewei@xidian.edu.cn (Z.R.); 2 National Demonstration Center of Experimental Teaching, Xidian University, Xi’an 710126, China; xdjackie@163.com; 3 Zhejiang Cachi New Energy Technology Co., Ltd., Huzhou 313100, China; yunmintang201111@163.com; 4 Beijing Institute of Nanoenergy and Nanosystems, Chinese Academy of Sciences, Beijing 101400, China; 5 School of Materials Science and Engineering, Georgia Institute of Technology, Atlanta, GA 30332-0245, USA

**Keywords:** miniaturized, self-powered, triboelectric nanogenerator, broad-frequency vibration energy

## Abstract

The widespread vibration is one of the most promising energy sources for IoT and small sensors, and broad-frequency vibration energy harvesting is important. Triboelectric nanogenerators (TENGs) can convert vibration energy into electrical energy through triboelectricity and electrostatic induction, providing an effective solution to the collection of broad-frequency vibration energy. Also, the power supply in constrained and compact spaces has been a long-standing challenge. Here, a miniaturized power supply (MPS) based on a broad-frequency vibration-driven triboelectric nanogenerator (TENG) is developed. The size of the MPS is 38 mm × 26 mm × 20 mm, which can adapt to most space-limited environments. The TENG device is optimized through theoretical mechanical modeling for the external stimuli, it can efficiently harvest vibrational energy in the frequency range of 1–100 Hz and has a high output power density of 134.11 W/cm^3^. The developed device demonstrates its practical application potential in powering small electronics like LEDs, watches, and timers.

## 1. Introduction

Wireless technologies, smart electronic devices, and clean and sustainable energy harvesting technologies are valuable for advancing the Internet of Things (IoT) [1,2,3]. Among different technologies for harvesting energy, the triboelectric nanogenerator (TENG) based on triboelectrification and electrostatic induction has sparked a great deal of interest [4,5]. On the one hand, the emerging TENG can convert mechanical energy from the surrounding environment into electrical energy, providing a new way of energy supply [6,7]. This approach is different from traditional energy sources and meets the needs of renewable and ubiquitous energy solutions. On the other hand, with flexibility, ease of fabrication, low cost, environmental suitability, and diverse material choices, and customized according to specific needs, the TENG devices demonstrate ideal application prospects in self-powered systems [8,9,10]. However, the recently reported TENGs are usually bulky, severely limiting their practicality in some application scenarios, especially in restrained spaces. This limitation underscores a critical requirement for power sources: they not only need to provide stable and continuous energy output but also have compact size characteristics and are easy to integrate. Against this background, there is an urgent need for optimization and miniaturization of the current TENG devices that can effectively harvest energy from the environment.

Vibration energy is one of the most promising energies for IoT due to its ubiquitous and readily available in the environment [11,12,13]. The TENG can convert these environmental vibration energies into electrical energy [14,15,16]. Therefore, harvesting vibration energy based on TENG provides a promising approach for self-powered systems within a limited space. Xu et al. proposed a TENG with a silicone rubber-spring helical structure [11]. The TENG effectively converted vibration energy from 0 to 30 Hz into electrical energy and served as a self-powered vibration sensor. Wang et al. prepared a TENG with a bidirectional two-degree-of-freedom structure, which harvested vibration energy of 1–25 Hz and continuously powered a stopwatch [17]. Yu et al. prepared a TENG with an additional mass-enhanced film structure [18]. The TENG effectively harvested the broadband vibration energy of 15–70 Hz and powered temperature and humidity sensors. However, there is still the problem of the limited frequency range of the collected ambient vibration energy. Most environmental vibrations have a relatively broad spectrum distribution, with a vibration frequency range of 1–100 Hz (the frequency range of human activities is 1–10 Hz, and the frequency range of vibration caused by machines is usually 10–100 Hz) [19]. Although the reported TENGs can work at both the frequency of human activities and the frequency of vibrations induced by machines, developing a TENG that can harvest the vibrational energy covering the broad vibration spectra from the environment is still an arduous task. Furthermore, realizing energy collection of a wide spectrum and device miniaturization significantly increases the complexity of the design and fabrication of the TENG devices. Generally, an effective self-powered system of miniaturized TENGs needs to meet the following conditions. First and foremost, the TENG can effectively work with a wide range of vibration frequencies in the environment instead of relying solely on one resonance frequency. Secondly, the output power of the TENG can reasonably power electronic devices. Then, the overall design of the devices needs to be miniaturized so that they can operate flexibly without being limited by space. Finally, the TENG can be effectively combined with a power management module (PMM) to achieve a self-powered system.

In this work, a miniaturized power supply (MPS) based on the broad-frequency vibration-driven TENG and PMM is developed. The size of the entire MPS is 38 mm × 26 mm × 20 mm, which can adapt to most space-limited environments. A mechanical model was established for the TENG, and the structural design of the MPS was optimized under different conditions such as mass, acceleration, and initial distance. The TENG in MPS can achieve an instantaneous output power of 2.65 mW and a high output power density of 134.11 W/cm^3^. The MPS can harvest most environmental vibrations with a frequency range of 1–100 Hz. Finally, it is demonstrated that MPS can power the LEDs, the watch, and the timer successfully. The successful exploration of MPS shows its great application prospects in miniaturized self-powered systems for harvesting vibration energy.

## 2. Materials and Methods

### 2.1. Fabrication of the MPS

The MPS mainly consists of a TENG and a PMM. In order to make the MPS compact in size and easy to integrate, the friction contact area of the TENG was selected to be 20 mm × 20 mm. Other components of the MPS were designed according to the area requirement. First, The framework of MPS was made by 3D printing (SLA 3D printer). The printing material is a UV-curable resin. The framework is a hollow cuboid (38 mm × 26 mm × 17 mm) without a top surface, with a thickness of 1.5 mm. The bottom surface of the framework was designed with four circular recesses positioned 2 mm from the bottom edge to accommodate the four springs (0.1 mm × 4 mm × 5 mm) of the TENG. The circular recesses have a diameter of 4 mm and a depth of 0.75 mm. The bottom surface also served as the base for the TENG. The aluminum (Al) film with a thickness of 60 μm and the fluorinated ethylene propylene (FEP) films with a thickness of 80 μm were pasted on the base and used as the electrode and the negative tribo-layer, respectively. Then, a solid board (32 mm × 20 mm × 1.5 mm) was 3D printed, and four circular recesses corresponding to the base were designed. The Al film was pasted on the board as an electrode and positive tribo-layer. All electrodes and friction layers are 20 mm × 20 mm. Subsequently, a piece of lead was fixed on the board to serve as the mass block. Finally, a printed circuit board (PCB) with a PMM was installed at the top of the framework. The dimensions of the PCB are 38 mm × 26 mm × 1.6 mm. Thus, the MPS was successfully fabricated. The size of the MPS is 38 mm × 26 mm × 20 mm.

### 2.2. Fabrication of the PMM

The PMM can divided into three parts: an alternating current (AC) to direct current (DC) conversion circuit, a self-management energy storage circuit, and a DC buck circuit. The following electronic components were used: rectifier bridge (DB107), capacitors (1000 pF and 47 uF), thyristor (EC103D1), Zener diode (1N4733), diode (MUR460), and H-shaped inductor (3.3 mH).

### 2.3. Measurement

External excitation is provided by a signal generator (AFG3252C, Tektronix, Beaverton, OR, USA), a power amplifier unit (HEA-200C, Nanjing, China), and a vibration platform. An electrometer (Keithley 6514, Cleveland, OH, USA) and a data acquisition module (USB-6356, NI, Shanghai, China) were used to measure the electrical performance of the MPS.

## 3. Results and Discussion

### 3.1. Design and Working Principle of the MPS

**Figure 1:**
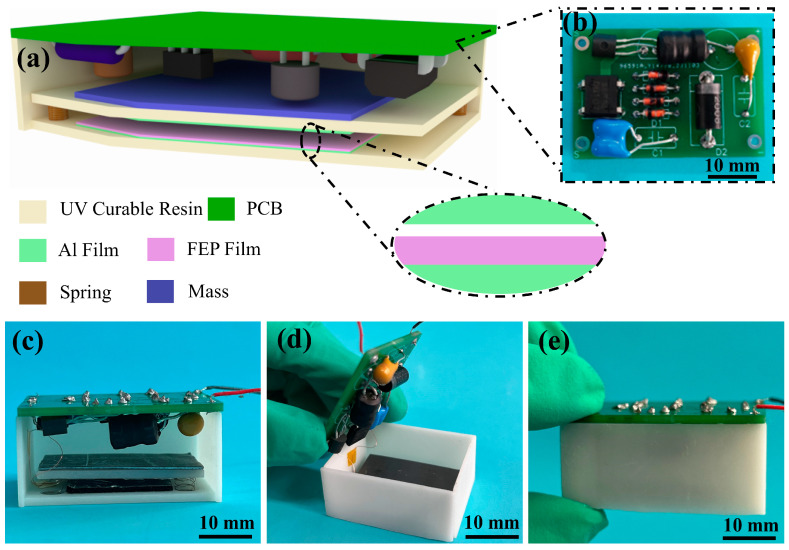
(**a**) Structure and materials design of MPS, inset is the illustration of the TENG unit; (**b**) photograph of the printed circuit board with a PMM; (**c**) front view of MPS; (**d**) top view of MPS; (**e**) photograph of the completely packaged MPS.

The structure and material design of the MPS are shown in Figure 1a. It can be seen that the MPS consists of a framework, a vibrational TENG, and a PCB with the PMM. It is worth noting that the framework is made from 3D printing, which does not require overly complicated assembly steps, facilitating mass production. A TENG unit is installed inside the framework, supported by four springs at the bottom, as shown in the inset in Figure 1a. The FEP film with good electron affinity attached to the bottom surface is the negative tribo-layer, and the Al film is selected as the electrode. Meanwhile, the Al film was pasted on the spring-supported board as an electrode and a positive tribo-layer. The board can move freely through the springs inside the framework, thus enabling the formation of a contact-separation type TENG with the framework’s base. Subsequently, a mass block is affixed to the board to facilitate vibration energy collection. The PCB with a PMM is placed upside down inside the framework to maximize space utilization, as shown in Figure 1b. The detailed preparation process and MPS materials are demonstrated in the experimental section. Figure 1c,d show the front and top views of the MPS. Figure 1e shows the completely packaged MPS. The entire system has a diminutive size, precisely 38 mm × 26 mm × 20 mm.

**Figure 2:**
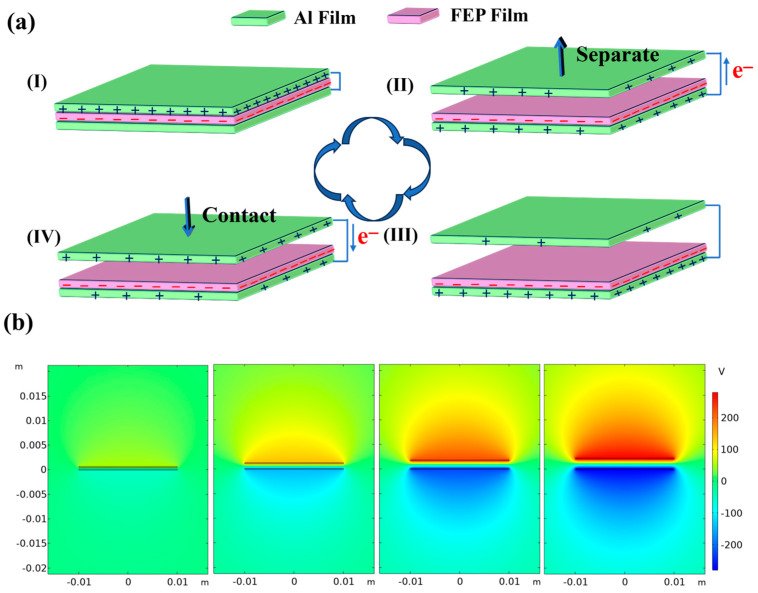
(**a**) Schematic illustrating the working mechanism of the TENG device; (**b**) COMSOL Multiphysics simulation results for the TENG.

The TENG is utilized by MPS to gather vibrational energy and convert it into electrical power. The working mechanism of the TENG is described in Figure 2. The electricity generation process of TENG can be divided into four stages, as illustrated by the TENG under a short-circuit condition in Figure 2a. Initially, under the stimulation of external vibrations, the spring’s oscillation induces mutual contact between the FEP membrane and the Al film (Ⅰ). The Al electrode and the FEP film have positive and negative charges because the FEP membrane is significantly more triboelectrically negative than the Al film [15,20,21,22]. Subsequently, under external mechanical vibrations, the Al membrane is separated from the FEP membrane, and the separated charges result in an electric potential between the two membranes. Because of the short-circuit connection and electrostatic induction, the electron flows from the bottom electrode to the top electrode (Ⅱ), so that the top electrode is less positively charged and the bottom electrode becomes more positively charged. When two membranes reach the maximum separation distance, the current flow reaches its end (III), while the electric potential difference attains its peak value when the TENG is under an open-circuit condition (not shown in the figure). Next, the Al film is pulled downward by the spring’s restoring force, and the electrons return to the bottom electrode, generating the opposite current (IV). A complete electricity generation process is formed until it returns to the initial position of contact (I). Electrons move bidirectionally between two electrodes, inducing alternating current in the external circuit. To further understand the potential change in TENG, COMSOL Multiphysics was used to perform finite element analysis to calculate the potential distribution between the two triboelectric layers, and the result is shown in Figure 2b. To facilitate calculation, two two-dimensional rectangles are used to represent the positive and negative electrodes of the TENG, respectively. It can be seen that the potential difference between the two electrodes increases as the distance between the electrodes gradually increases. The simulation outcomes corroborate the working mechanism shown in Figure 2a.

### 3.2. Mechanical Model and Optimization of the MPS

**Figure 3:**
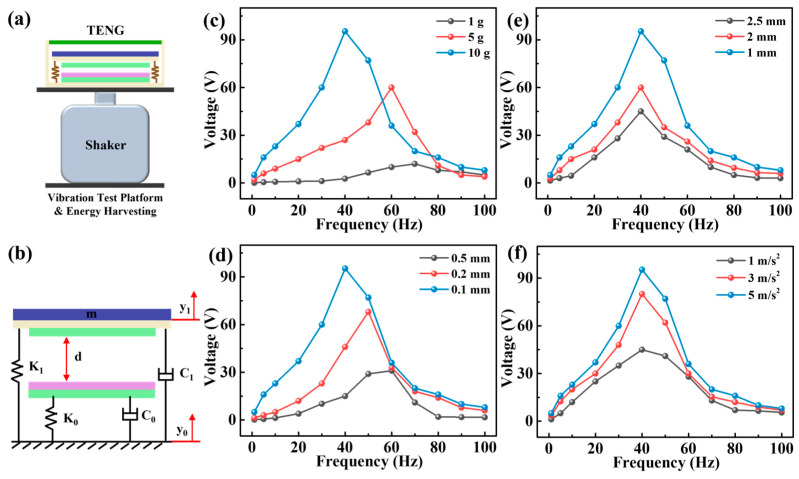
(**a**) Test device for vibration energy harvesting; (**b**) mechanical model of TENG in MPS; (**c**) output response of the TENG when changing (**c**) moving masses, (**d**) wire diameter of the springs, (**e**) initial distances and (**f**) excitation accelerations.

The frequency responses of the TENG in MPS were tested by placing the device on a vibration platform, as shown in Figure 3a. The testing system comprises an accelerometer sensor, a vibration control system, and a data acquisition system. Optimization of the TENG is deemed essential to collect vibration energy more efficiently. Therefore, a TENG mechanical model was established using a single-degree-of-freedom spring-mass-damper, as demonstrated in Figure 3b [23,24,25]. Then, the dynamics of TENG can be expressed by the following differential equation.

(1) $$m{\ddot{y}}_{1}+c_{1}{\dot{y}}_{1}+k_{1}y_{1}-F_{e}=-m{\ddot{y}}_{0},(y_{1}>-d)$$

(2) $$m{\ddot{y}}_{1}+{(c_{0}+c}_{1}{)\dot{y}}_{1}+(k_{0}+k_{1}{)y}_{1}+k_{0}d=-m{\ddot{y}}_{0},(y_{1}\leq -d)$$

where *y*_0_(*t*) = *Ysin*(*ωt*), *Y* is the excitation amplitude at the base, ω is the excitation frequency, *y*_0_ is the excitation of the base, *y*_1_ is its relative motion, *d* is the initial distance, *F_e_* is the electrostatic force, and *m* is the block mass. The relative motion of the model can be divided into two stages. When the relative motion of *m* is less than the *d*, the stiffness and damping of the entire system are *k*_0_ and *c*_0_, respectively. When the relative motion of *m* exceeds d, the stiffness and damping of the entire system are *k*_0_ *+ k*_1_ and *c*_0_ + *c*_1_, respectively. To better study the frequency characteristics of TENG, we use the averaging method to solve the differential Equations (1) and (2). The specific procedure of the derivation is described in detail in the Supporting Information [24,26,27]. The following conclusion can be drawn by solving Equations (1) and (2): the frequency characteristics of TENG are related to mass, stiffness, initial distance, and acceleration. Consequently, the design of TENGs is optimized by studying the electrical output under different parameter conditions. When changing the parameters, the fixed mass, acceleration, wire diameter of spring, and initial distance are 10 g, 5 m/s^2^, 0.1 mm, and 1 mm, respectively. As demonstrated in Figure 3c, different masses significantly influence the open-circuit voltage (*V_OC_*) output and frequency bandwidth of the TENG. The *V_OC_* and frequency bandwidth increase as the mass increases. This is probably attributed to the greater mass, as the contact and separation of the two friction layers are more thorough. Furthermore, the natural frequency of the TENG is determined as follows [28,29]:

(3) $$f=\frac{1}{2\pi }\sqrt{\frac{k}{m}}$$

where *k* is the spring stiffness and *m* is the mass, respectively. This implies that the natural frequency is inversely proportional to mass. Therefore, the natural frequency of TENG shifts towards a lower frequency. The *k* is determined as follows [28,29]:

(4) $$k=\frac{Gd^{4}}{8D_{2}^{3}}$$

where *G* is the shear modulus, *d* is the wire diameter of the spring, and *D* is the diameter of the spring, respectively. It can be concluded that the stiffness of the spring is related to the wire diameter (other parameters remain unchanged) and has a proportional relationship. Therefore, the influence of different wire diameters is discussed. As shown in Figure 3d, the bandwidth became greater with the smaller wire diameter. That is because when the spring stiffness is reduced, the natural frequency of the TENG decreases, resulting in an expanded effective response frequency range of the system. Additionally, the *V_OC_* became greater with the smaller wire diameter. A smaller spring stiffness results in a softer spring. TENG requires less force to produce larger deformations, thereby achieving larger amplitudes [24]. The greater the amplitude then generates the greater the electrical output. The effects of different initial distances on TENG are presented in Figure 3e. It can be observed that as the initial distance decreases, both the bandwidth and the output increase, because the smaller the distance, the greater the force and the larger the contact area. In addition, the increase in acceleration can increase the contact area between the two friction layers, thereby increasing the electrical output and bandwidth of the TENG, as shown in Figure 3f. The results suggest that increasing the mass, reducing the stiffness and initial distance, and increasing the excitation acceleration are effective ways to improve the output and bandwidth of TENG.

### 3.3. Output Performances of the TENG in MPS

**Figure 4:**
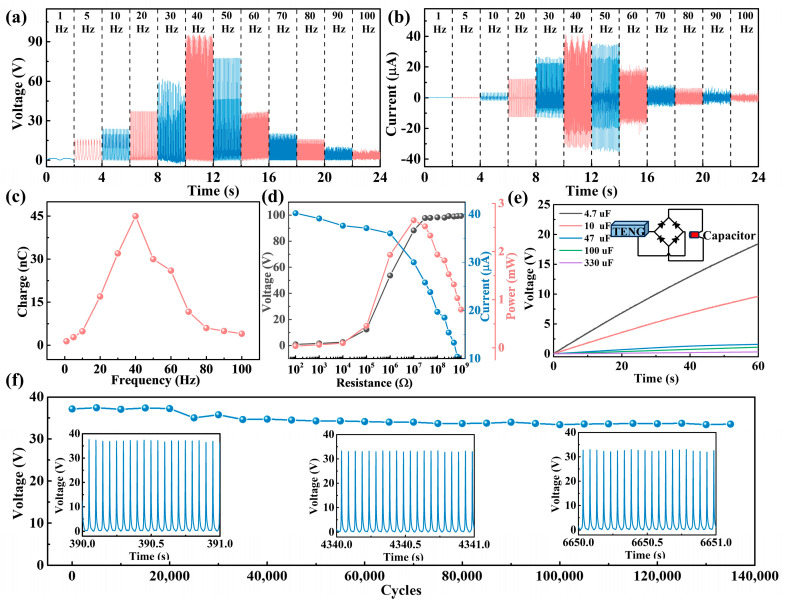
The output (**a**) *V_OC_*, (**b**) *I_SC_* and (**c**) QSC of the TENG in MPS at the vibration frequency of 1–100 Hz; (**d**) output power and (**e**) demonstration of charging a capacitor of the TENG at the vibration frequency of 40 Hz; (**f**) cycling stability testing at a frequency of 20 Hz.

From the abovementioned model and experimental analysis, the acceleration of 5 m/s^2^, the initial distance of 1 mm, the wire diameter of 0.1 mm, and the mass of 10 g were finally selected for harvesting environmental vibration energy in a wider range. Subsequently, we conducted a systematic study on the collection of vibration energy at different frequencies by the TENG (Figure 4). As shown in Figure 4a–c, the TENG can harvest vibration frequencies ranging from 1 to 100 Hz. With the increase in frequency, it can be observed that the output (*V_OC_*, short-circuit current (*I_SC_*), and transferred charge (*Q_SC_*)) of the TENG exhibit a trend of initially increasing and then decreasing. When the frequency is 40 Hz, the output is maximized, with a *V_OC_* of 95.3 V, an *I_SC_* of 41.1 μA, and a *Q_SC_* of 45 nC. This is because, at 40 Hz, the resonance frequency of the TENG is reached, resulting in the maximum contact area and contact force between the two friction layers. Various resistors were employed as external loads to investigate the output characteristics of the TENG further. As shown in Figure 4d, the TENG achieves an instantaneous output power of 2.65 mW and a power density of 134.11 W/cm^3^ under a matching resistance of 10 MΩ. Table 1 compares the MPS in this study with the previously reported small-sized TENG. Compared to previously reported designs [11,21,30,31,32,33,34], the proposed MPS exhibits a smaller volume, higher power density, and broader operating frequency range. The charging response of the TENG to various capacitors is presented in Figure 4e. A schematic of the test can be found in the inset. Smaller capacitors possess a faster charging rate. The TENG can charge 4.7, 10, 47, 100, and 330 μF capacitors to 18.4, 9.7, 1.6, 1.1, and 0.4 V within 60 s, respectively. This indicates that TENG possesses excellent power supply capabilities. Furthermore, TENG’s output stability was tested to validate its power supply capabilities further, as depicted in Figure 4f. It can be noted that the output of TENG can still maintain 90.3% after 135,000 cycles at a frequency of 20 Hz. The results suggest that the TENG demonstrates good stability in its performance, indicative of its reliable operational characteristics. However, at 40 Hz, the output of TENG can only remain at 51.6% after 144,000 cycles (Figure S1a, Supporting Information). The curve of the output VOC also became unstable during the cycle test (Figure S1b–d, Supporting Information). This is because the amplitude of the device will increase significantly at the resonant frequency. It may cause device structural fatigue, cracks or other forms of structural damage during long-term operation, thereby reducing the overall stability and life of the device [35,36].

### 3.4. Potential Application of the MPS

**Figure 5:**
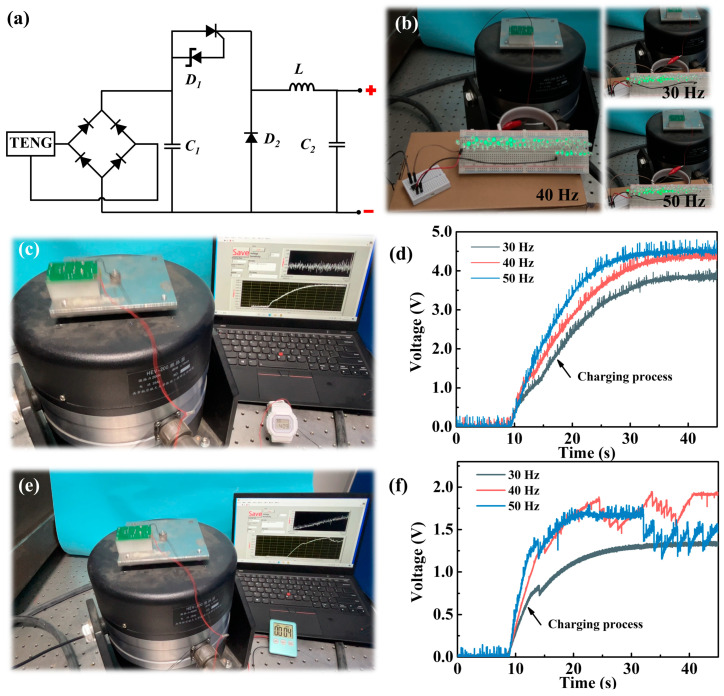
(**a**) Circuit schematic of the PMM; (**b**) photograph of MPS lighting 70 LED lights at different frequencies; (**c**) photograph of the watch powered by MPS at 40 Hz; (**d**) voltage variation in the watch at different frequencies; (**e**) photograph of the timer powered by MPS at 40 Hz; (**f**) voltage variation in the timer at different frequencies.

The TENG can power small electronic devices. As shown in Figure 5b, 70 LED lights can be lit up at different frequencies. Specifically, 70 LED lights can be lit up at a frequency of 40 Hz, verifying the practicality of the TENG. To harvest environmental vibrational energy more effectively and realize a self-powering management system, constructing the miniature self-powering device requires integrating a miniature energy management circuit with the TENG. Therefore, a micro-printed circuit board with a common PMM was installed at the top of the framework to build the MPS. The size of the entire MPS is 38 mm × 26 mm × 20 mm, which can adapt to most space-limited environments. The circuit schematic diagram for PMM is depicted in Figure 5a, which consists of the rectifier bridge, capacitors, thyristor, Zener diode, diode, and H-shaped inductor [16,37,38]. Firstly, the output of the TENG is converted from AC to DC through a rectifier bridge. The TENG charges capacitor *C*_1_ through a direct current (DC) supply. When the voltage of *C*_1_ exceeds the voltage of the Zener diode (*D*_1_), the threshold switch composed of the *D*_1_ and the thyristor will be activated. Then, the diode (*D*_2_) allows the LC circuit, which is composed of an inductor (*L*) and a capacitor (*C*_2_), to oscillate half a cycle. Once activated, the LC circuit converts magnetic field energy into low electric field energy. Due to the effect of *L*, when *C*_2_ releases the charge, an induced current is generated in *L*. Then, a reverse voltage will be generated in the circuit, enhancing the charge in *C*_2_. In this way, high voltage can be converted into low voltage, and a small amount of charges can be converted into a large amount of charges through the PMM. The output of the TENG can autonomously operate the PMM.

Further validation of MPS application prospects can be achieved by illuminating the timer and the watch. Figure 5c shows that the watch can be lit up by MPS at 40 Hz. To verify that it can work in a wider range, it was also tested in the frequency range of 30–50 Hz, as shown in Figure 5d. The results show that the watch can be charged to 3.8, 4.4, and 4.5 V with frequencies of 30, 40, and 50 Hz, respectively. It is worth noting that the watch only needs to be illuminated at 1.5 V, indicating that the MPS can power the watch continuously. The timer can also be powered by MPS, as shown in Figure 5e. Furthermore, the voltage rapidly increases and remains constant when the timer is continuously operated in the frequency range of 30–50 Hz (Figure 5f). The above results show that MPS can operate in a wide frequency range, and has great potential in vibration energy generated in large mechanical equipment such as compressors, pumps, etc., as well as vehicles and railway systems. It also demonstrates the great application prospects in miniaturized self-powered vibration energy harvesting systems.

## 4. Conclusions

In summary, the MPS based on the broad-frequency vibration-driven TENG with the PMM is proposed. The size of the entire MPS is 38 mm × 26 mm × 20 mm, which can adapt to most space-limited environments. To optimize the MPS, a mechanical model of the TENG was established, and the frequency response of the TENG under different conditions, such as mass, acceleration, and initial distance, was studied in detail. The results show that the output and bandwidth of TENG increase significantly with mass and acceleration. The output of TENG increases as the initial distance and wire diameter of the spring decrease. The TENG in MPS can achieve an instantaneous output power of 2.65 mW. The MPS is optimized to harvest most environmental vibrations with a 1–100 Hz frequency range. Finally, it is demonstrated that MPS can successfully power 70 LEDs, the watch, and the timer. However, MPS also faces several challenges, including improving energy conversion efficiency, improving energy storage and management systems, and enhancing environmental adaptability. Despite these challenges, the successful exploration of MPS shows great development prospects in areas such as wearable devices, the IoT, smart city development, and environmental monitoring. With continuous research and innovation, MPS technology is expected to become an important branch in the field of energy harvesting and conversion in the future.

## Figures and Tables

**Table 1:** Comparison between the proposed miniaturized TENGs.

Devices	Volume (cm^3^)	Power Density (W/m³)	Frequency Range (Hz)	Ref.
MPS	19.67	134.11	1–100	This work
Elastic multiunit TENG	44.46	102	2–30	[21]
VTENG	48	0.90	1–100	[30]
HSI-TENG	25	50	10–60	[31]
C-TENG	44.3	23.4	5–15	[32]
CH-TENG	37.67	3.84	10–50	[33]
S-TENG	54	4.0	0–30	[11]
H-TENG	448	8.26	5–25	[34]

## Data Availability

All of the relevant data are included in this published article.

## Supplementary Materials

The following supporting information can be downloaded at: https://www.mdpi.com/article/10.3390/mi15050645/s1, Figure S1: (a) Cycling stability testing of the TENG in MPS at a frequency of 40 Hz; (b–d) the output *V_OC_* of the TENG in MPS during cyclic stability testing process.
