# Supplementary material for: Miniaturized and High Volumetric Energy Density Power Supply Device Based on a Broad-Frequency Vibration Driven Triboelectric Nanogenerator
Source: Micromachines (Basel). 2024 May 13;15(5):645. doi: 10.3390/mi15050645 (PMC11123006; doi:10.3390/mi15050645)
Supplement: Supplementary file 1 [file micromachines-15-00645-s001.zip › micromachines-2934484-supplementary.pdf]

Miniaturized and high volumetric energy density power supply device based on a broad-frequency vibration driven triboelectric nanogenerator

Liting Wu [1], Zewei Ren [1], Yanjun Wang [2], Yumin Tang [3], Zhong Lin Wang [4,5,*] and Rusen Yang [1,*]

[1] School of Advanced Materials and Nanotechnology, Xidian University, Xi'an 710126, China
[2] National Demonstration Center of Experimental Teaching, Xidian University, Xi'an 710126, China
[3] Zhejiang Cachi New Energy Technology CO., Ltd, Huzhou 313100, China
[4] Beijing Institute of Nanoenergy and Nanosystems, Chinese Academy of Sciences, Beijing 101400, China
[5] School of Materials Science and Engineering, Georgia Institute of Technology, Atlanta, GA 30332-0245, USA
* Correspondence: zhong.wang@mse.gatech.edu (Z.W.); rsyang@xidian.edu.cn (R.Y.)

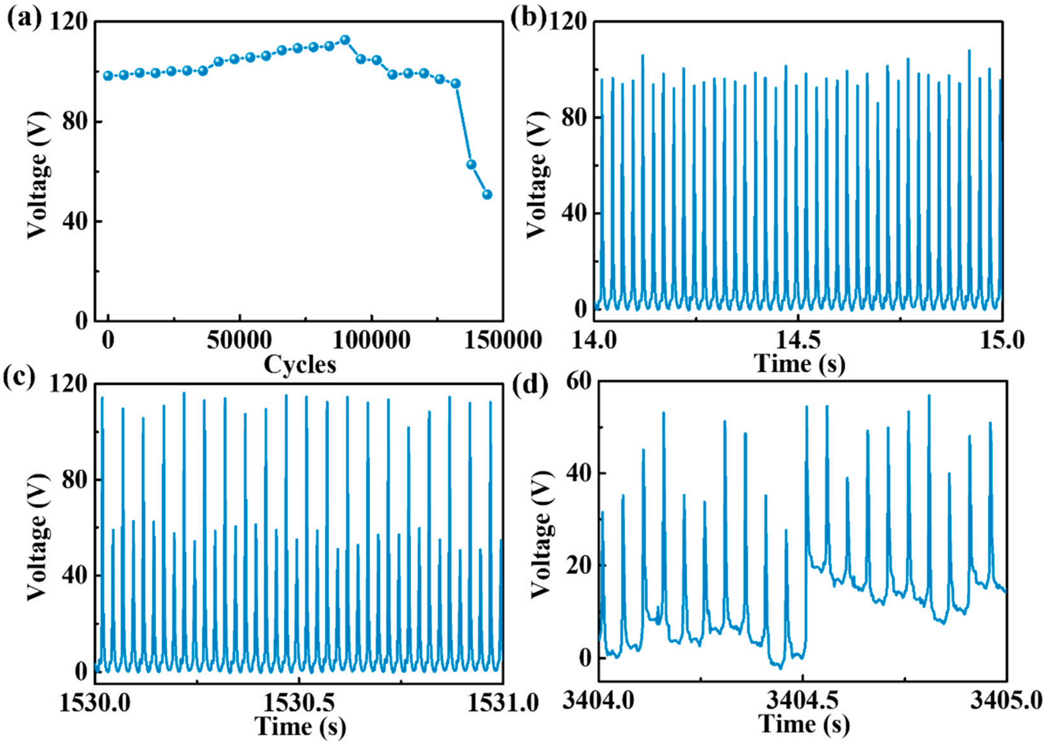

Figure S1. (**a**) Cycling stability testing of the TENG in MPS at a frequency of 40 Hz; (**b-d**) the output V_{OC} of the TENG in MPS during cyclic stability testing process.

Supporting Notes

The electrostatic force between the two friction layers is much smaller than the elastic force and that can be ignored. According to Figure 3b, the formulas can be rearranged as follows [24,26,27]:

$$\ddot{y}_1 + 2\xi_1\omega_1\dot{y}_1 + \omega_1^2 y_1 = -\ddot{y}_0, (y_1 > -d) \quad \text{(S1)}$$

$$\ddot{y}_1 + (2\xi_0\omega_0 + 2\xi_1\omega_1)\dot{y}_1 + (\omega_0^2 + \omega_1^2)y_1 + \omega_0^2 d = -\ddot{y}_0, (y_1 \le -d) \quad \text{(S2)}$$

where $y_0(t) = Ysin(\omega t)$, Y is the excitation amplitude at the base, ω is the excitation frequency, y_0 is the excitation of the base, y_1 is its relative motion, d is the initial distance, ξ_1 and ω_1 are the suspension damping and angular frequency of Al friction layer, and ξ_0 and ω_0 are the suspension damping and angular frequency of FEP friction layer, which can be further defined as $2\xi_1\omega_1 = \frac{c_1}{m}$, $2\xi_0\omega_0 = \frac{c_0}{m}$, $\omega_1^2 = \frac{k_1}{m}$, $\omega_0^2 = \frac{k_0}{m}$. To study the frequency response of the TENG, a dimensionless variable is used:

$$\tau = \omega_1 t, \rho = \frac{\omega}{\omega_1}, \rho_1 = \frac{\omega_0}{\omega_1}, u = \frac{y_1}{Y}, v = \frac{y_0}{Y} = sin(\rho\tau), \dot{u} = \frac{du}{d\tau}, \dot{v} = \frac{dv}{d\tau}, \delta = \frac{d}{Y} \quad \text{(S3)}$$

We can obtain

$$\dot{y}_1 = \omega_1 Y\dot{u}, \dot{y}_0 = \omega_1 Y\dot{v} \quad \text{(S4)}$$

$$\ddot{u} + 2\xi\dot{u} + u = \rho^2 sin(\rho\tau) + f(u, \dot{u}) \quad \text{(S5)}$$

where

$$f(u,\dot{u}) = \begin{cases} 0, u > -\delta \\ -2\xi_1\rho_1\dot{u} - \rho_1^2 u - \rho_1^2\delta, u \le -\delta \end{cases} \quad \text{(S6)}$$

By using the averaging method, the first-order approximate solution of (S5) is assumed to be:

$$u = a(\tau)\, sin(\varphi(\tau)) \quad \text{(S7)}$$

$$\dot{u} = a(\tau)\rho\, cos(\varphi(\tau)) \quad \text{(S8)}$$

$$\varphi(\tau) = \rho\tau + \beta(\tau) \quad \text{(S9)}$$

where $a(\tau)$ is a slowly varying amplitude, and $\beta(\tau)$ is a slowly varying phase difference between the base excitation and the amplitude response. A conclusion can be obtained by Equations (S7) and (S8).

$$\dot{a}\, sin\,\varphi + a\dot{\beta}\, cos\,\varphi = 0 \quad \text{(S10)}$$

Substituting Equations (S7) and (S8) into (S5) obtains

$$\dot{a}\rho\, cos\,\varphi - a\dot{\beta}\rho\, sin\,\varphi = a(\rho^2 - 1)\, sin\,\varphi + \rho^2 sin(\varphi - \beta) - 2\xi a\rho\, cos\,\varphi + f(u,\dot{u}) \quad \text{(S11)}$$

Solving Equations (S10) and (S11) for $\dot{a}$ and $\dot{\beta}$, it has

$$\dot{a}\rho = [a(\rho^2 - 1) + \rho^2 cos\,\beta]\, sin\,\varphi\, cos\,\varphi - (2\xi a\rho + \rho^2 sin\,\beta)\, cos^2\varphi + f(u,\dot{u})\, cos\,\varphi \quad \text{(S12)}$$

$$a\dot{\beta}\rho = -[a(\rho^2 - 1) + \rho^2 cos\,\beta]\, sin^2\varphi + (2\xi a\rho + \rho^2 sin\,\beta)\, cos\,\varphi\, sin\,\varphi - f(u,\dot{u})\, sin\,\varphi \quad \text{(S13)}$$

Since the variables $\dot{a}$ and $\dot{\beta}$ vary slowly, it may suppose that their average values remain constant over a cycle period of 2π:

$$2\pi\dot{a}\rho = \int_0^{2\pi}[a(\rho^2 - 1) + \rho^2 cos\,\beta]\, sin\,\varphi\, cos\,\varphi\, d\varphi + \int_0^{2\pi}[-(2\xi a\rho + \rho^2 sin\,\beta)\, cos^2\varphi + f(u,\dot{u})\, cos\,\varphi]\, d\varphi \quad \text{(S14)}$$

$$2\pi a\dot{\beta}\rho = \int_0^{2\pi}[-a(\rho^2 - 1) - \rho^2 cos\,\beta]\, sin^2\varphi\, d\varphi + \int_0^{2\pi}(2\xi a\rho + \rho^2 sin\,\beta)\, cos\,\varphi\, sin\,\varphi\, d\varphi - \int_0^{2\pi} f(u,\dot{u})\, sin\,\varphi\, d\varphi \quad \text{(S15)}$$

where

$$f(u,\dot{u}) = \begin{cases} 0, (0 < \varphi < \pi + \varphi_1, 2\pi - \varphi_1 < \varphi < 2\pi) \\ -2\xi_1\rho_1 a\rho \cos\varphi - \rho_1^2 a \sin\varphi - \rho_1^2\delta, (\pi + \varphi_1 < \varphi < 2\pi - \varphi_1) \end{cases} \tag{S16}$$

$\varphi_1 = \sin^{-1}(\delta/a)$ is the phase values when the proof mass engages the up-plate.

For the steady-state response solution of the system, the time derivatives on the left-hand sides of Equations (S14) and (S15) are considered into zero. Hence, integration of Equations (S14) and (S15) gives:

$$\pi\rho^2 \sin\beta = -2\xi_1 a\rho\pi - \rho_0\xi_0 a\rho(\pi - 2\varphi_1 - \sin 2\varphi_1) \tag{S17}$$

$$\pi\rho^2 \cos\beta = \pi a(1-\rho^2) - \begin{bmatrix} -\frac{1}{2}\rho_0^2 a(\pi - 2\varphi_1 + \sin 2\varphi_1) \\ +2\rho_0^2\delta\cos\varphi_1 \end{bmatrix} \tag{S18}$$

Combining Equations (S17) and (S18), the implicit Equation for the amplitude a as a function of the excitation frequency ρ is given by

$$\pi^2\rho^4 = Z_1^2 + Z_2^2 \tag{S19}$$

where Z_1 and Z_2 are in the right-hand sides of Equations (S17) and (S18), respectively. Based on the frequency response function (S19), the dimensionless amplitude a with respect to frequency ρ can be obtained accordingly.

References:

[24] Qi, Y., Liu, G., Gao, Y., Bu, T., Zhang, X., Xu, C., Lin, Y., Zhang, C. Frequency Band Characteristics of a Triboelectric Nanogenerator and Ultra-Wide-Band Vibrational Energy Harvesting. *ACS Appl. Mater. Interfaces* **2021**, *13*, 26084-26092.

[26] Liu H., Lee C., Kobayashi T., Tay C.J., Quan C. Investigation of a MEMS piezoelectric energy harvester system with a frequency-widened-bandwidth mechanism introduced by mechanical stoppers. *Smart Mater. Struct.* **2012**, *21*, 035005.

[27] Chen J., Zhu G., Yang W., Jing Q., Bai P., Yang Y., Hou T.C., Wang Z.L. Harmonic-resonator-based triboelectric nanogenerator as a sustainable power source and a self-powered active vibration sensor. *Adv. Mater.* **2013**, *25*, 6094-6099.
